# Transcatheter aortic valve replacement associated infective endocarditis case series: broadening the criteria for diagnosis is the need of the hour

**DOI:** 10.1186/s12872-021-02364-0

**Published:** 2021-11-20

**Authors:** Kriti Lnu, Shamim Ansari, Shantanu Mahto, Hemal Gada, Mubashir Mumtaz, David Loran, Nikhil J. Theckumparapil, Amit N. Vora

**Affiliations:** 1 Medical Center Pinnacle, University of Pittsburgh, 111 S. Front St, Harrisburg, PA 17101 USA; 2 Jawaharlal Institute of Postgraduate Medical Education and Research, Pondicherry, India

**Keywords:** TAVR, Infective endocarditis, Transcatheter aortic valve replacement, Complications, Prosthetic valve endocarditis, Multi-modal imagining

## Abstract

**Background:**

Transcatheter valve replacement (TAVR) is an important therapeutic intervention for patients with aortic valve stenosis. As TAVR has become available to a broader population, there has been an increase in the number of less common, yet potentially catastrophic, complications. TAVR related infective endocarditis (TAVR-IE) is a rare, but potentially fatal, complication.

**Case series:**

We present here two patients that we encountered for TAVR associated infective endocarditis. Our first patient presented 5 weeks after his TAVR. His initial presentation was consistent with signs of sepsis. The patient then developed Mobitz type I block during hospital course. His TEE was negative for features of infective endocarditis. Due to high suspicion, patient was taken for surgical exploration and was found to have multiple foci of vegetation adhered to the stent frame. Our second patient presented with new onset pulmonary edema, worsening heart failure and systemic inflammatory response. A TEE was done for persistent MSSA bacteremia which showed stable prosthetic valve function with no signs of infective endocarditis. Patient was discharged with a prolonged course of intravenous antibiotics. Patient was re-admitted for worsening sepsis and blood cultures were positive for MSSA. Patient was taken for surgical exploration of his prosthetic aortic valve which showed purulent aortic root abscess.

**Conclusion:**

Through these cases, we aim to raise awareness on TAVR-IE. Due to the atypical clinical presentation, the modified Duke criteria may not be sufficient to diagnose TAVR-IE. Transesophageal echocardiogram in TAVR-IE may be negative or indeterminate. Prosthetic valve shadow may obscure smaller vegetations and/or smaller abscesses. A negative transesophageal echocardiogram should not rule out TAVR-IE and further diagnostic imaging modalities should be considered. PET/CT after administration of 18F-FDG (fluorodeoxyglucose) is a useful diagnostic tool in the diagnosis of infective endocarditis where TEE has been negative or inconclusive. Multi-modal imaging, in addition to the modified Duke criteria, can facilitate early diagnosis and improved mortality outcomes.

**Supplementary Information:**

The online version contains supplementary material available at 10.1186/s12872-021-02364-0.

## Introduction

The emergence of transcatheter valve replacement (TAVR) has revolutionized the treatment and care of patients with symptomatic severe aortic stenosis (AS) across the spectrum of risk. With increasing clinical expertise and the advent of a new generation of valves, outcomes have improved significantly and TAVR is now being implemented in a much broader spectrum of patients than ever before.

With TAVR becoming available to a larger population, we also now see a rise in complications that may be less common yet catastrophic. Some of the grave complications of TAVR include paravalvular leak, stroke, vascular injury, heart block, and prosthetic valve endocarditis (PVE). TAVR-IE is a rare, but potentially fatal, complication. One of the challenges in the management of such cases is timely diagnosis. Patients with IE may fail to meet the modified Duke criteria [1–4]. Transesophageal echocardiogram (TEE), which remains an important diagnostic option for diagnosis in native valve endocarditis, may yield negative or inconclusive results in patients with prosthetic valves. This could be due to the higher density of the prosthetic valve or the metallic frame, which can cause impedance in the ultrasonic waves and acoustic shadowing. As such, a multi-modal imaging approach should be adopted in these patients [5].

We present two cases of TAVR-IE. These patients had negative TEE for apparent vegetation and failed to meet the modified Duke criteria for IE. Through these case reports and the literature review, we aim to raise the suspicion for TAVR-IE in order to facilitate timely diagnosis and to prevent adverse outcomes.

### Case 1

**Fig. 1:**
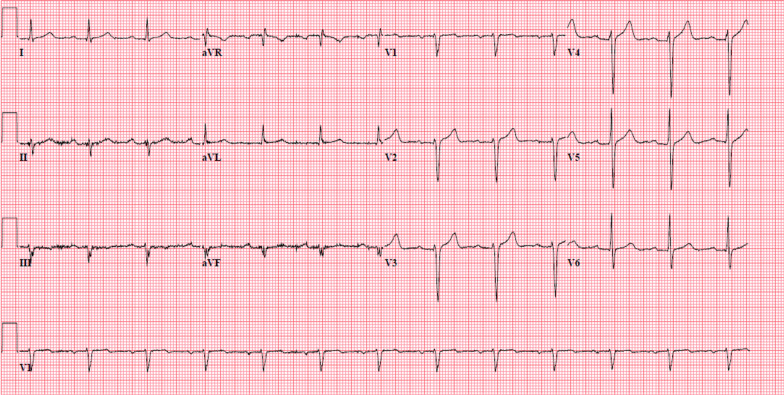
Baseline EKG on admission

**Fig. 2:**
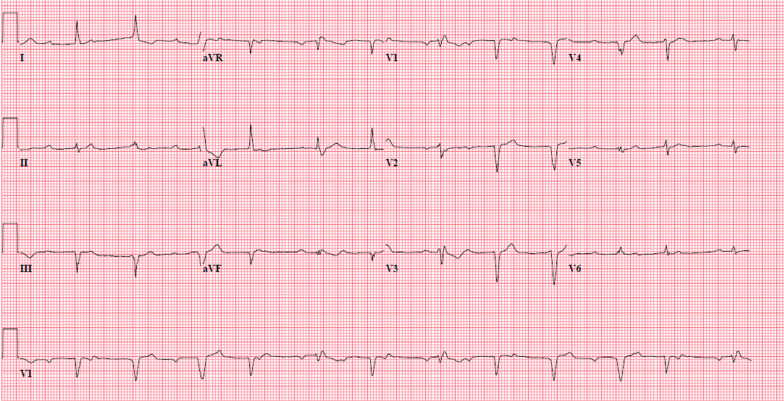
Sinus rhythm with 2nd degree A-V block (mobitz I) with premature ventricular complexes or fusion complexes

A 79-year-old male (Table 1) presented with altered mental status and back pain 5 weeks after undergoing TAVR with a 29 mm Medtronic Corevalve Evolut Pro (Medtronic, Galway, Ireland) via transfemoral approach. He had been seen in the outpatient clinic 3 days earlier and no clinical changes, EKG or transthoracic echocardiogram (TTE) abnormalities were noted at that time. In the emergency department, the patient was found to be tachycardiac with heart rate of 110 bpm, tachypneic with respiratory rate of 32 and hypoxic with SpO_2_ of 96% on 2 L O_2_ via nasal cannula. Lab results showed: white blood cell count of 5.5 K/μL with 31% bands, lactic acid of 2.2 mmol/L and a normal comprehensive metabolic panel. Urine analysis was negative for any abnormality. CT brain did not show any acute intracranial abnormality and contrast enhanced CT scan of the abdomen and pelvis was unremarkable for any acute process. He was started empirically on broad spectrum antibiotics with vancomycin 15 mg/kg/day and cefepime 1 g every 6 h. Initial blood cultures showed gram positive cocci. MRI of spine was performed which was negative for infection. On day 2, the patient developed Mobitz type I block, widened QRS, and wide escape beats of varying morphology (Figs. 1, 2).

**Table 1:** Clinical features of the patients

	Past medical history	Aortic Stenosis Severity			Perioperative antibiotic prophylaxis at TAVR	Post op course	1 month post TAVR Echocardiogram
		Aortic valve area (cm2)	Mean pressure Gradient (Mm Hg)	Peak velocity (m/sec)			
Patient 1	Type 2 Diabetes mellitus, Hyperlipidemia, Hypertension	0.84	51.8	44.6	Yes	No complication	EF: 60–65% Mean gradient:12 mm Hg Aortic valve area: 2 cm^2^
Patient 2	Hypertension, Hyperlipidemia, Non-obstructive coronary artery disease, systolic and diastolic heart failure, paroxysmal atrial fibrillation	0.79	68.7	53.9	Yes	No complication	EF: 40–45% (improved from before of 30–35%) Mean gradient: 8.7 Mm Hg Aortic valve area: 2.4 cm^2^

Blood cultures showed methicillin-sensitive Staphylococcus aureus (MSSA). The patient underwent TEE, which showed thickening in the posterior aspect of the aortic root near the site of the interatrial septum. It also showed increased paravalvular aortic regurgitation when compared to the post-TAVR echocardiogram. However, there was no clear evidence of vegetation.

The initial plan was to perform a tagged white blood scan, however due to unavailability of the scan, the patient’s worsening conditioning, and high suspicion for TAVR associated endocarditis, the patient was taken for surgical exploration (Additional file 1: Video S1, Additional file 2: Video S2, Additional file 3: Video S3).

**Fig. 3:**
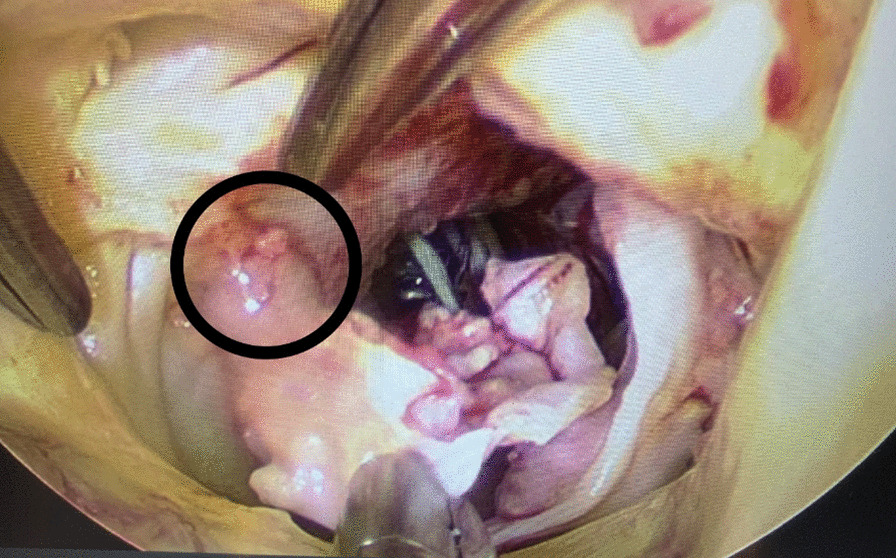
Abscessed area between the right and left coronary cusps

The explanted valve had multiple foci of vegetation adherent to the stent frame. There was aortic root abscess. The prosthetic valve was replaced with a 27 mm Magna-Ease valve (Edwards Lifesciences Corp. Irvine, CA) (Additional file 4: Video S4) (Fig. 3).

The patient was discharged with antibiotics on day 14 in stable condition. Aspirin and clopidogrel were continued at discharge. Patient showed a well-functioning valve with no clinical symptoms at 6 months follow up (Additional file 8).

### Case 2

A 71-year-old male with (Table 1) presented with generalized weakness, altered mental status, abdominal pain, diarrhea and fever 8 weeks after TAVR with a 34 mm Corevalve Evolut Pro. He had been seen in the outpatient clinic 2 weeks earlier. No clinical or echocardiographic changes were noted from a previous post-TAVR echocardiogram. In the emergency department, the patient was found to have a temperature of 37.8 C, heart rate of 111 beats per minute, respiratory rate of 36 breath per minute and saturating 92% on room air with normal blood pressure. Complete blood count showed normal leucocyte count with 21% bandemia and platelet count of 86,000/microliter which was chronic for the patient. His basic metabolic panel results were significant for bicarbonate level of 13.6 mEq/L with a lactic acid level of 6.6 mmol/L. Liver function test and coagulation profile was normal on admission. Initial EKG did not show any new changes from the baseline. Chest X-ray did not show any evidence of acute process. Contrast-enhanced CT scan of the abdomen and pelvis did not show any pathology. Infectious panel was sent for diarrhea which included clostridium difficile, and patient was started empirically on broad spectrum antibiotics with vancomycin 15 mg/kg/day, cefepime 1 g every 6 h and metronidazole 500 mg daily. A TTE was done which showed stable prosthetic valve function with no new regurgitation. The patient continued to remain febrile and tachypneic with altered mental status on broad spectrum antibiotics. Initial blood culture showed gram positive cocci in pairs and clumps in 4/4 bottles. A repeat TEE was done which showed no evidence of valvular vegetation; however, the aortic valve bioprosthesis was suboptimally visualized. A trace degree of aortic insufficiency was noted. Blood cultures resulted in MSSA bacteremia in 4/4 bottles and antibiotics were deescalated to intravenous cefazolin (Additional file 5: Video S5, Additional file 9).

One week after presentation the patient developed polymorphic ventricular tachycardia and fibrillation requiring CPR/defibrillation. He also developed acute interstitial nephritis from cefazolin requiring intermittent hemodialysis and was switched to vancomycin/rifampin. The patient continued to improve hemodynamically and clinically, and given no clear evidence of infective endocarditis he was managed medically. Blood cultures became negative and after 9 days and he was discharged to acute rehab with a wearable cardioverter/defibrillator, and his aspirin and apixaban were continued at discharge.

In rehab, the patient developed persistent fever and patient was re-admitted for developing sepsis. Blood cultures were again positive for MSSA. At this time, a decision was made for explantation of the prosthetic aortic valve based on clinical suspicion of endocarditis.

**Fig. 4:**
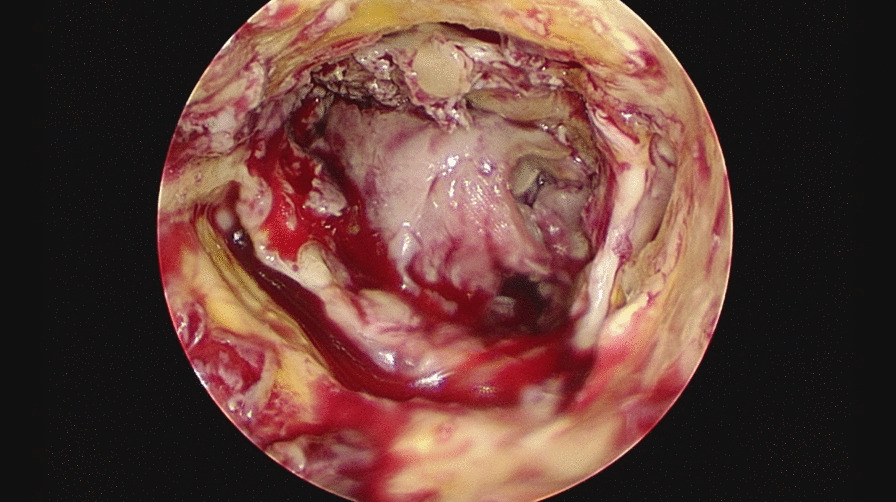
Aortic valve endocarditis with purulent root abscess

**Fig. 5:**
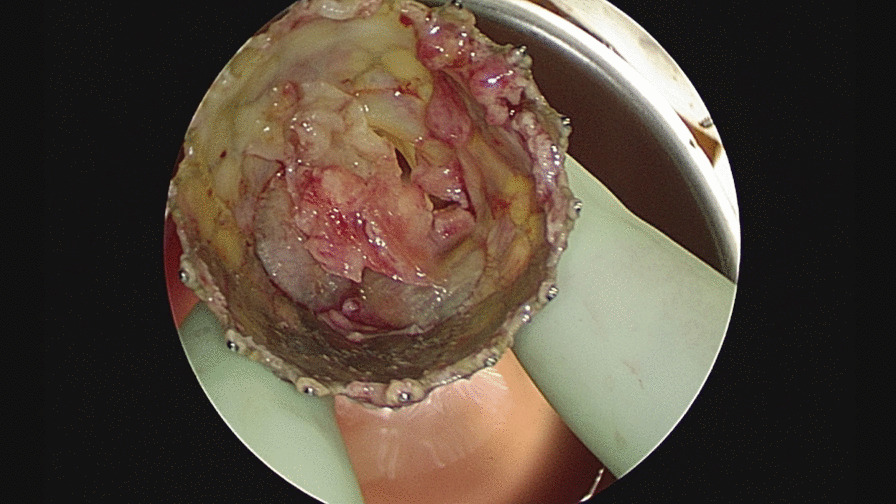
Explanted aortic valve with purulent root abscess

The explanted valve showed purulent root abscess. A 25 mm Intuity valve (Edwards Lifesciences Corp. Irvine, CA, USA) with Cardiocel pericardial patch (LeMaitre Vascular, Inc., Burlington MA, USA) was placed between the non-coronary cusp and right coronary cusp (Figs. 4, 5).

The patient developed hemodynamic instability and bedside emergent extracorporeal membrane oxygenation (ECMO) was deployed. He developed gastrointestinal ischemia, renal failure and acute blood loss while on ECMO. Due to his poor prognosis, the patient's family requested comfort care.

## Discussion

With technological advances, newer generation valves, and increasingly favorable outcomes, TAVR is an indispensable procedure in interventional cardiology. With advancing use, we also see a broader spectrum of complications that may not be adequately diagnosed with strict criteria. Both of our patients failed to meet modified Duke’s criteria for IE which delayed diagnosis as well as inadvertently required unnecessary investigations. Through this literature review, we aim to increase the knowledge regarding TAVR-IE to prevent catastrophic complications.

### Incidence

TAVR has become well established as the procedure of choice across all surgical risk patients. With the increasing number of procedures being performed, secondary complications have become an emerging cause of morbidity and mortality. TAVR-IE remains a rare complication.

The incidence of TAVR-IE ranges from 0.1 to 4.4% [1, 6, 7]. The Placement of Aortic Transcatheter Valves (PARTNER II) trial estimates the incidence of infective endocarditis after TAVR at 1.44% [8] but the literature shows the incidence to be up to 4.4% [7], with no difference between TAVR and surgical aortic valve replacement (SAVR).

TAVR-IE can be categorized as early (< 2 months), intermediate (2 months to 1 year), or late (> 1 year) based on the time of diagnosis post implantation. The incidence rates are reported to be 18% for early, 62% for intermediate, and 20% for late TAVR-IE [9, 10]. The risk of endocarditis was greatest in the first year after TAVR [9]. Our patients both presented less than 90 days after TAVR.

### Risk factors

Multiple risk factors have been associated with increased risk of TAVR-IE. The risk factors can be grouped as modifiable and non-modifiable. Non modifiable risk factors include young age [2, 3, 5] and male sex [3, 7, 11, 12].

Modifiable factors include chronic kidney disease, higher BMI [13] and diabetes mellitus [3, 6, 7]. Liver cirrhosis, pulmonary disease, peripheral artery disease and chronic dialysis have also been identified as independent risk factors for TAVR-IE [13]. Our review of 16 case reports (Additional file 6: Table S1, Additional file 7) found that that 10 out of the 16 patients described in the reports were male. Major comorbidities reported in these cases include diabetes mellitus, chronic kidney disease and chronic obstructive pulmonary disease. Society of Thoracic Surgeons Predicted Risk of Mortality (PROM) scores of 8 or higher are associated with a higher risk of infective endocarditis in TAVR patients [13].

Nosocomial infections have also been identified as risk factors in TAVR patients as they can act as source of seeding [14]. We noted 4 patients in the literature in whom nosocomial infection could have been a potential cause for TAVR-IE; one patient had undergone a dental procedure, the second had a history of total parenteral nutrition, the third had undergone surgery—a high anterior resection, and the fourth had a history of repeated hospital admissions.

Mangner et al. and Reguiro et al., in their studies determined that moderate or severe para-aortic regurgitations were a significant risk factor for PVE [3, 6]. Mangner also stated that valve-in-valve or more than one prosthetic valve carried a higher risk for IE post TAVR.

The literature does not show any differences in the occurrence of TAVR-IE between procedures performed in the operating room versus those done in a catheterization lab [15]. According to Amat-Santos et al., orotracheal intubation increased the risk of TAVR-IE. They also showed that the self-expandable CoreValve (Medtronic Inc., MN, USA) seemed to illustrate a higher risk when compared to balloon expanded valve [2]. In our review of the case reports (Additional file 6: Table S1, Additional file 7), TAVR-IE was associated with three CoreValves, 5 balloon expandable valves, 3 bioprosthetic valves, and 1 other valve. Valve type was not reported in 3 cases.

### Microbiology

The most common microorganisms known to cause TAVR-IE are gram-positive cocci, which include Staphylococcus aureus, coagulase negative staphylococci, and enterococci [1–3, 6, 16–18]. Streptococcus has also been reported to be an important cause of TAVR-IE [19].

Other reported causal organisms associated with TAVR related IE include gram negative rods: E. coli [16, 17, 20], acinetobacter [2, 21] pseudomonas aeruginosa [18, 22], serratia [2], and salmonella [21]. Our patients both had MSSA bacteremia.

Our review of case reports shows a variety of pathogens associated with TAVR-IE: Staphylococcus aureus (1 case), Streptococcus (5 cases), Enterococcus (3 cases), and atypical organisms Moraxella (1 case), Corynebacterium (2 cases), and Candida (2 cases). In two cases, no organisms were isolated.

TAVR-IE shows a different pathogen alignment than SAVR. Enterococcus has a higher incidence in TAVR than SAVR [23]. This could be due to the transfemoral approach, as intertriginous regions such as the groin can harbor enterococcus, which grows better in warm, humid conditions.

### Clinical features

The most common presenting feature of patients with TAVR-IE is fever, which was seen in both of our patients. Both patients presented with altered mental status, which could be secondary to severe sepsis.

The second most common initial feature seen in patients with TAVR-IE is clinical heart failure, irrespective of previous systolic and diastolic dysfunction [1, 6]. Our first patient did not have a history of diastolic or systolic heart failure. On presentation, he was found to have pulmonary congestion with dyspnea, BNP of 1490, and his ejection fraction was reduced to 50–55% from 60 to 65%. The second patient also showed a newly decreased ejection fraction, in this case, to 40%.

Another possible complication is new conduction defects or arrhythmias. Our first patient developed a new Mobitz type I block with wide QRS, raising concern for aortic root abscess. He further deteriorated, developed Mobitz type II block and required transvenous pacing. The second patient developed episodes of polymorphic ventricular tachycardia and, on day 3, developed ventricular fibrillation requiring CPR and defibrillation. He subsequently developed tachy-brady syndrome, further complicating management.

Clinical presentations may include, but are not limited to, newly developed heart block, arrhythmias, and embolic phenomenon, with stroke as the most common, and signs and symptoms of source infection, if present [24]. Constitutional symptoms may be present in patients with severe sepsis.

### Diagnosis

The modified Duke criteria remains the cornerstone of diagnosis of IE. The criteria incorporate clinical findings, echocardiographic evidence and are supported by blood cultures, serological markers and bio-pathological evidence.

As discussed above, patients with TAVR-IE may have atypical symptoms that are not included in the modified Duke criteria. Echocardiographic findings may be negative or inconclusive. TEE in TAVR-IE may be negative or indeterminate. Prosthetic valve shadow may obscure smaller vegetations and smaller abscesses. This is because of the higher density of the prosthetic valve, which impedes the passage of ultrasound waves. The metallic and polymeric components of both mechanical and biologic valves do not transmit ultrasound waves and can lead to sub-optimal imaging due to acoustic shadowing. It may also be difficult to assess the severity and location of paravalvular aortic regurgitation with TAVR-IE because the anatomy and physiology of regurgitant jets differ from those in conventional valves.

Many cases in literature have shown patients who had TAVR-IE but had negative or inconclusive TEE [1–4]. For both of our patients, infective endocarditis was considered a high probability and TEE was done. TEE for the first patient showed moderately increased aortic regurgitation but no evidence of aortic root abscess or vegetation. The second patient had a well-functioning valve with trace aortic insufficiency and no evidence of vegetation or abscess.

Hence, although considered gold standard of diagnosis in IE related to native valve, TEE may be inconclusive in TAVR-IE and a negative TEE should not be used to rule out IE post TAVR [24, 25].

### New diagnostic modalities

Multiple diagnostic modalities have been enumerated by the European Society of Cardiology for diagnosis of IE in prosthetic valves [5]. These include multi-slice CT, contrast enhanced multi-slice CT and ^18^F-FDG PET/CT (PET/CT after administration of fluorodeoxyglucose). This is emerging as a useful diagnostic tool in the diagnosis of infective endocarditis when TEE is negative or inconclusive [4, 26].

^18^F-FDG PET/CT is a relatively safe and non-invasive procedure with no absolute contraindications. It is safe to use in patients with renal failure, contrast allergy, and in patients with implantable cardiac devices. ^18^F-FDG is taken up in tissues with infection and inflammation and hence can focally tag infectious processes in the cardiac tissues. It can also show the extent of valve infection as well as extra-cardiac manifestations of IE.

^18^F-FDG PET/CT has shown higher efficacy than TEE in diagnosing prosthetic valve endocarditis [27, 28]. One meta-analysis showed a sensitivity of 80.5% when using ^18^F-FDG PET/CT in patients with prosthetic valve IE [29]. Another study showed that when analyzed specifically for IE in prosthetic valve, ^18^F-FDG PET/CT had 85% sensitivity [30]. A recent meta-analysis by Wang et al. [31] showed a pooled sensitivity of 0.86 (0.81–0.89, 60.0%) when the ^18^F-FDG PET/CT was analyzed for the sub-group of prosthetic valve endocarditis. Saby et al. showed that abnormal uptake of ^18^F-FDG in addition to the modified Duke criteria showed an increase in sensitivity of diagnosing prosthetic valve IE to up to 97% [32]. These studies indicate the usefulness of the PET/CT scan in patients with prosthetic valve endocarditis. The sensitivity of testing in patient’s irrespective of meeting modified Duke’s criteria is considerably high and can prevent catastrophic complications due to delay in diagnosis. Per European Society of Cardiology guidelines [5], ^18^F-FDG PET/CT should be considered in all patients with suspected PVE who have an inconclusive or negative TEE. However, this is currently limited to patients who received the prosthesis at least 3 months prior. This is a limitation as TAVR-IE usually presents early as discussed above.

### Management and outcome

The mortality rate associated with TAVR-IE has been reported to be as high as 46% [24]. Complications include aortic root dissection, paravalvular and aortic root abscesses, intra/paravalvular regurgitation, stroke, and high rates of heart failure. A study showed a higher incidence of aortic root destruction when the time between symptom development and diagnosis was more than 14 days [33].

Patients presenting with TAVR-IE should be managed with a multi-disciplinary team including cardiology, cardiothoracic surgery, infectious disease and internal medicine. Timely diagnosis and early initiation of antibiotics, with possible surgery, may prevent catastrophic and fatal outcomes [2, 24]. While surgery with valve explantation is the definitive treatment of TAVR-IE [11, 13], many patients who have undergone TAVR have high surgical risk to begin with, and the risk further increases with IE. Hemodynamic instability may further preclude any surgical intervention and hence many patients are initially managed conservatively with a prolonged course of intravenous antibiotics. Broad spectrum antibiotics are recommended empirically as patients may not have typical microorganisms as the causative factor.

## Conclusion

Through our case reports, we aim to raise awareness regarding the atypical symptoms that patients may present. A patient with previous TAVR may present with symptoms that do not meet the modified Duke’s criteria for diagnosis of infective endocarditis. The symptoms of fever, new heart failure, conduction defects, new arrhythmias and/or bacteremia with atypical organism, should raise high concern for IE. A negative TEE may result in delayed diagnosis and management. The addition of multi-modal imaging as an alternate to echocardiogram in the modified Duke’s criteria can help in early diagnosis. Per the ESC 2015 guidelines, ^18^F-FDG PET/CT is recommended as an alternate major criterion to TEE. However, this is limited to patients who had a prosthesis implanted at least 3 months prior and hence does not apply to a significant sub-set of the patients with TAVR-IE. Recent literature does show increased sensitivity of ^18^F-FDG PET/CT in all prosthetic valve endocarditis patients and should be considered in all patients who fail to meet modified Duke’s criteria.

Prosthetic aortic valve endocarditis carries high mortality and hence clinical suspicion should always supersede set criteria. There is a need for multi-center registries for TAVR-IE to broaden the scope for accurate and efficient diagnosis.

## Supplementary Information

**Additional file 1: Video S1.** There is a TAVR present. There is normal leaflet excursion without obvious vegetations. There is no obvious peri-aortic abscess. There is mild to moderate paravalvular insufficiency present.

**Additional file 2: Video S2.** There is a TAVR present. There is normal leaflet excursion without obvious vegetations. There is no obvious peri-aortic abscess. There is mild to moderate paravalvular insufficiency present.

**Additional file 3: Video S3.** There is a TAVR present. There is normal leaflet excursion without obvious vegetations. There is no obvious peri-aortic abscess. There is mild to moderate paravalvular insufficiency present.

**Additional file 4: Video S4.** Endocarditis seen in the CoreValve struts.

**Additional file 5: Video S5.** There is a bioprosthetic aortic valve present. Visualization of the bioprosthetic aortic valve was difficult. No vegetations were noted. There is no aortic insufficiency present.

**Additional file 6: Table S1.**

**Additional file 7.** TAVR Table references.

**Additional file 8.** CARE checklist for Case 1.

**Additional file 9.** CARE checklist for Case 2.

## Data Availability

Not applicable.

## Abbreviations

TAVR: Transcatheter aortic valve replacement
IE: Infective endocarditis
TAVR-IE: Transcatheter aortic valve replacement associated infective endocarditis
TEE: Transesophageal echocardiogram
MSSA: Methicillin sensitive staphylococcus aureus
PET/CT with 18F-FDG: Positron emission tomography scan/computed tomography scan with administration of 18- fluorodeoxyglucose
PVE: Prosthetic valve endocarditis
TTE: Transthoracic echocardiogram
EKG: Electrocardiogram
CPR: Cardiopulmonary resuscitation
ECMO: Extracorporeal membrane oxygenation
TAVI: Transcatheter aortic valve implantation
PARTNER II: Placement of aortic transcatheter valves trial
BMI: Body mass index
SAVR: Surgical aortic valve replacement
