# Supplementary material for: Transcatheter aortic valve replacement associated infective endocarditis case series: broadening the criteria for diagnosis is the need of the hour
Source: BMC Cardiovasc Disord. 2021 Nov 20;21:559. doi: 10.1186/s12872-021-02364-0 (PMC8606088; doi:10.1186/s12872-021-02364-0)
Supplement: Supplementary file 7 — Additional file 7. TAVR Table references. [file 12872_2021_2364_MOESM7_ESM.docx]

References for Table in order they appear :

1 - Gürtler N, Osthoff M, Rueter F, Wüthrich D, Zimmerli L, Egli A, et al. Prosthetic valve endocarditis caused by Pseudomonas aeruginosa with variable antibacterial resistance profiles: a diagnostic challenge. BMC Infect Dis. 2019 Jun 17;19(1):530.

2- Ruchonnet EP, Roumy A, Rancati V, Kirsch M. Prosthetic Valve Endocarditis after Transcatheter Aortic Valve Implantation Complicated by Paravalvular Abscess and Treated by Pericardial Patches and Sutureless Valve Replacement. Heart Surg Forum. 2019 04 4;22(2):E155-E158.

3- Amat-Santos IJ, Cortés C, Varela-Falcón LH. Delayed left anterior mitral leaflet perforation and infective endocarditis after transapical aortic valve implantation-Case report and systematic review. Catheter Cardiovasc Interv. 2017 Apr;89(5):951-4.

4- Ahmad K, Klaaborg KE, Hjortdal V, Nørgaard BL, Terkelsen CJ, Jensen K, et al. Prosthetic valve endocarditis after transcatheter aortic valve implantation-diagnostic and surgical considerations. J Thorac Dis. 2016 Oct;8(10):E1213-E1218.

5- Moufarrej R, Aljaberi N. Prosthetic valve endocarditis secondary to Corynebacterium following transcatheter aortic valve implantation: a case report. Eur Heart J Case Rep. 2018 Dec;2(4):yty109.

6- Sarı C, Durmaz T, Karaduman BD, Keleş T, Bayram H, Baştuğ S, et al. Prosthetic valve endocarditis 7 months after transcatheter aortic valve implantation diagnosed with 3D TEE. Hellenic J Cardiol. 2016 Mar-Apr;57(2):119-23.

7- Elghannam M, Haldenwang PL, Aljabery Y, Moustafine V, Strauch J. Open transcatheter aortic valve implantation on a degenerated bioprosthetic valve conduit. J Surg Case Rep. 2018 Sep;2018(9):rjy233.

8- Fathi AS, Ali JM, Mann S, Taghavi J, Davies WR, Sudarshan C. Emergency valve-in-valve transcatheter aortic valve implantation for endocarditis degeneration. J Card Surg. 2020 Mar;35(3):713-5.

9- Carrel T, Eberle B. Candida Endocarditis after TAVR. N Engl J Med. 2019 Jan 3;380(1):e1.

10- Morioka H, Tokuda Y, Oshima H, Iguchi M, Tomita Y, Usui A, et al. Fungal endocarditis after transcatheter aortic valve replacement (TAVR): Case report and review of literature. J Infect Chemother. 2019 Mar;25(3):215-7.

11- Naqvi SY, Salama IG, Narins C, Stuver T. Corynebacterium striatum prosthetic valve endocarditis with severe aortic regurgitation successfully treated with transcatheter aortic valve replacement. BMJ Case Rep. 2018 Nov 28;11(1):e226881

12- Olsthoorn JR, Lam K, Verberkmoes NJ. Endocarditis after transcatheter aortic valve replacement; a new nightmare in cardiac surgery. J Card Surg. 2019 Nov;34(11):1420-1.

13- Zhigalov K, Khokhlunov M, Szczechowicz M, Mashhour A, Mkalaluh S, Easo J, et al. Right Anterior Minithoracotomy for Endocarditis After Transcatheter Aortic Valve Replacement. Ann Thorac Surg. 2020 01;109(1):e17-e19.

14- González YO, Ung R, Blackshear JL, Laman SM. Three-Dimensional Echocardiography for Diagnosis of Transcatheter Prosthetic Aortic Valve Endocarditis. CASE (Phila). 2017 Aug;1(4):155-8.

15- Nguyen C, Cheong AP, Himbert D. Valve-in-valve-in-valve: Treating endocarditis of a transcatheter heart valve. Catheter Cardiovasc Interv. 2015 Oct;86(4):E200-4.

16- Rafiq I, Parthasarathy H, Tremlett C, Freeman LJ, Mullin M. Infective endocarditis caused by Moraxella nonliquefaciens in a percutaneous aortic valve replacement. Cardiovasc Revasc Med. 2011 May-Jun;12(3):184-6.
