# Supplementary material for: Transcatheter aortic valve replacement associated infective endocarditis case series: broadening the criteria for diagnosis is the need of the hour
Source: BMC Cardiovasc Disord. 2021 Nov 20;21:559. doi: 10.1186/s12872-021-02364-0 (PMC8606088; doi:10.1186/s12872-021-02364-0)
Supplement: Supplementary file 9 — Additional file 9. CARE checklist for Case 2. [file 12872_2021_2364_MOESM9_ESM.pdf]

CARE case report guidelines

CARE Checklist of information to include when writing a case report

(cc) BY-NC-ND

Topic	Item	Checklist item description	Reported on Line
Title	1	The diagnosis or intervention of primary focus followed by the words “case report”	Page 1
Key Words	2	2 to 5 key words that identify diagnoses or interventions in this case report, including "case report"	Page 2
Abstract (no references)	3a	Introduction: What is unique about this case and what does it add to the scientific literature?	Page 2 background
	3b	Main symptoms and/or important clinical findings	Page 2 cases
	3c	The main diagnoses, therapeutic interventions, and outcomes	Page 2 cases
	3d	Conclusion—What is the main “take-away” lesson(s) from this case?	Page 2 -3 conclusion
Introduction	4	One or two paragraphs summarizing why this case is unique (**may include references**)	Page 3 : bacground
Patient Information	5a	De-identified patient specific information.	Page 6
	5b	Primary concerns and symptoms of the patient.	Page 6
	5c	Medical, family, and psycho-social history including relevant genetic information	Page 6
	5d	Relevant past interventions with outcomes	Page 6
Clinical Findings	6	Describe significant physical examination (PE) and important clinical findings.	Page6
Timeline	7	Historical and current information from this episode of care organized as a timeline	Page 6
Diagnostic Assessment	8a	Diagnostic testing (such as PE, laboratory testing, imaging, surveys).	Page 6
	8b	Diagnostic challenges (such as access to testing, financial, or cultural)	not applicable
	8c	Diagnosis (including other diagnoses considered)	Page 7
	8d	Prognosis (such as staging in oncology) where applicable	Page 7
Therapeutic Intervention	9a	Types of therapeutic intervention (such as pharmacologic, surgical, preventive, self-care)	Page 7
	9b	Administration of therapeutic intervention (such as dosage, strength, duration)	Page 7
	9c	Changes in therapeutic intervention (with rationale)	not applicable
Follow-up and Outcomes	10a	Clinician and patient-assessed outcomes (if available)	not applicable
	10b	Important follow-up diagnostic and other test results	Page 7
	10c	Intervention adherence and tolerability (How was this assessed?)	not applicable
	10d	Adverse and unanticipated events	not applicable
Discussion	11a	A scientific discussion of the strengths AND limitations associated with this case report	not applicable
	11b	Discussion of the relevant medical literature **with references**.	Page 8
	11c	The scientific rationale for any conclusions (including assessment of possible causes)	Page 8
	11d	The primary “take-away” lessons of this case report (without references) in a one paragraph conclusion	Page 14
Patient Perspective	12	The patient should share their perspective in one to two paragraphs on the treatment(s) they received	not applicable
Informed Consent	13	Did the patient give informed consent? Please provide if requested	**Yes** ☑ **No** ☐
